# Association Between Bariatric Surgery and Major Adverse Diabetes Outcomes in Patients With Diabetes and Obesity

**DOI:** 10.1001/jamanetworkopen.2021.6820

**Published:** 2021-04-26

**Authors:** Aristithes G. Doumouras, Yung Lee, J. Michael Paterson, Hertzel C. Gerstein, Baiju R. Shah, Branavan Sivapathasundaram, Jean-Eric Tarride, Mehran Anvari, Dennis Hong

**Affiliations:** 1 Division of General Surgery, McMaster University, Hamilton, Canada; 2 ICES, Toronto, Canada; 3 Institute of Health Policy, Management, and Evaluation, University of Toronto, Toronto, Canada; 4 Population Health Research Institute, Hamilton, Canada; 5 Department of Medicine, McMaster University, Hamilton, Canada; 6 Department of Medicine, University of Toronto, Toronto, Canada; 7 Sunnybrook Research Institute, Toronto, Canada; 8 Programs for Assessment of Technology in Health Research Institute, St Joseph’s Healthcare, McMaster University, Hamilton, Canada; 9 Department of Clinical Epidemiology and Biostatistics, Faculty of Health Sciences, McMaster University, Hamilton, Canada

## Abstract

**Importance:**

There are high-quality randomized clinical trial data demonstrating the effect of bariatric surgery on type 2 diabetes remission, but these studies are not powered to study mortality in this patient group. Large observational studies are warranted to study the association of bariatric surgery with mortality in patients with type 2 diabetes.

**Objective:**

To determine the association between bariatric surgery and all-cause mortality among patients with type 2 diabetes and severe obesity.

**Design, Setting, and Participants:**

This retrospective, population-based matched cohort study included patients with type 2 diabetes and body mass index (BMI; calculated as weight in kilograms divided by height in meters squared) 35 or greater who underwent bariatric surgery from January 2010 to December 2016 in Ontario, Canada. Multiple linked administrative databases were used to define confounders, including age, baseline BMI, sex, comorbidities, duration of diabetes diagnosis, health care utilization, socioeconomic status, smoking status, substance abuse, cancer screening, and psychiatric history. Potential controls were identified from a primary care electronic medical record database. Data were analyzed in 2020.

**Exposure:**

Bariatric surgery (gastric bypass and sleeve gastrectomy) and nonsurgical management of obesity provided by the primary care physician.

**Main Outcomes and Measures:**

The primary outcome was all-cause mortality. Secondary outcomes were cause-specific mortality and nonfatal morbidities. Groups were compared through a multivariable Cox proportional Hazards model.

**Results:**

A total of 6910 patients (mean [SD] age at baseline, 52.04 [9.45] years; 4950 [71.6%] women) were included, with 3455 patients who underwent bariatric surgery and 3455 match controls and a median (interquartile range) follow-up time of 4.6 (3.22-6.35) years. In the surgery group, 83 patients (2.4%) died, compared with 178 individuals (5.2%) in the control group (hazard ratio [HR] 0.53 [95% CI, 0.41-0.69]; *P* < .001). Bariatric surgery was associated with a 68% lower cardiovascular mortality (HR, 0.32 [95% CI, 0.15-0.66]; *P* = .002) and a 34% lower rate of composite cardiac events (HR, 0.68 [95% CI, 0.55-0.85]; *P* < .001). Risk of nonfatal renal events was also 42% lower in the surgical group compared with the control group (HR, 0.58 [95% CI, 0.35-0.95], *P* = .03). Of the groups that had the highest absolute benefit associated with bariatric surgery, men had an absolute risk reduction (ARR) of 3.7% (95% CI, 1.7%-5.7%), individuals with more than 15 years of diabetes had an ARR of 4.3% (95% CI, 0.8%-7.8%), and individuals aged 55 years or older had an ARR of 4.7% (95% CI, 3.0%-6.4%).

**Conclusions and Relevance:**

These findings suggest that bariatric surgery was associated with reduced all-cause mortality and diabetes-specific cardiac and renal outcomes in patients with type 2 diabetes and severe obesity.

## Key Points

**Question:**

Is there an association between bariatric surgery and all-cause mortality in patients with type 2 diabetes and severe obesity?

**Findings:**

In this cohort study that included 6910 patients with type 2 diabetes and severe obesity, there was a 47% decrease in all-cause mortality, a 68% decrease in cardiovascular mortality, and a 34% decrease in composite cardiac events associated with bariatric surgery compared with a nonsurgical control group after 4.6 years of follow-up. Patients who underwent surgery also had a 42% decrease in nonfatal renal events.

**Meaning:**

These findings suggest that bariatric surgery was associated with substantially lower all-cause mortality as well as nonfatal diabetic events in patients with type 2 diabetes.

## Teaser

This cohort study examines the association of bariatric surgery with all-cause mortality among individuals with diabetes and obesity compared with a matched nonsurgical control group.

## Introduction

Global rates of obesity and type 2 diabetes are increasing together at a rapid pace, with the prevalence of obesity increasing 2-fold in 73 countries since 1980, and approximately 23% of individuals who are morbidly obese have diabetes.^1,2^ Furthermore, obesity is associated with increased mortality with up to 20 years of life lost, and most obesity-associated mortality can be attributed to diabetes and cardiovascular causes.^3^ In individuals with obesity and diabetes, bariatric surgery is known to result in remission of comorbidities in 50% to 70% of individuals.^4,5,6^ Thus, the impact of surgery on overall mortality and the macrovascular complications in individuals with diabetes is of great importance, especially considering pharmacological and weight management therapies have relatively modest effects on mortality.^7,8,9^

While randomized clinical trial have established that bariatric surgery is an effective treatment for diabetes in individuals with obesity and can result in remission of diabetes, hypertension, and other cardiovascular risk factors, there is limited randomized clinical trial evidence reporting mortality and other important long-term cardiovascular outcomes in these individuals owing to the duration of follow-up.^10,11,12^ To bridge this gap, early observational studies have demonstrated mortality benefits associated with bariatric surgery, and incremental improvements in methods have been made over time to strengthen this evidence.^13^ Some gaps from previous studies have been that they did provide within-strata information on the association of surgery with mortality outcomes or consider potential confounding factors, such as cancer screening, substance use, or psychiatric history, all of which have been previously associated with mortality.^14,15^

Therefore, we performed a multicenter population-based cohort study that matched patients with obesity and diabetes who underwent bariatric surgery with a nonsurgical control group. Using data from multiple linked administrative databases, we matched individuals on a comprehensive list of confounders to determine the association of bariatric surgery with mortality and complications of diabetes.

## Methods

This cohort study was approved by the Hamilton Integrated Research Ethics Board. As data for this study were collected from ICES databases, informed consent was waived. ICES is a prescribed entity for the purposes of section 45 Ontario’s Personal Health Information Privacy Act (PHIPA). This means that health information custodians are permitted to disclose personal health information about their patients to ICES without consent. This information is for statistical analysis to evaluate and monitor aspects of the health system. ICES may also use personal health information under the authority of PHIPA for approved research projects. Data custodians outside the health sector may disclose personal information to ICES for specified use under the authority of Freedom of Information and Protection of Privacy Act (FIPPA) or other data-governing statutes. This study followed the Strengthening the Reporting of Observational Studies in Epidemiology (STROBE) reporting guideline for cohort studies.

### Overview of Study Design

This population-based matched cohort study used multiple linked databases that included patients with diabetes who were eligible for bariatric surgery within a primary care practice and received bariatric surgery or routine care for diabetes and obesity. Patients were excluded if they would have been deemed ineligible for surgery. The index date was the date of surgery for the exposed group and the date of the primary care physician appointment at which eligibility could be verified for the unexposed group. All confounders and matching variables would have been defined previous to the index dates.

### Setting

This study was completed in the province of Ontario, Canada. All residents of Ontario have access to bariatric surgery through a centralized online referral process. This referral process is generally completed by the individual’s primary care physician and is based on National Institutes of Health criteria. Patients are then equally distributed based on proximity to the nearest Center of Excellence, which is governed by the Ontario Bariatric Network.^16^ All hospitals within the Ontario Bariatric Network undergo accreditation and must complete more than 125 bariatric surgeries, and each surgeon must perform more than 50 bariatric surgeries per year.^17^ In Ontario, gastric bypass accounts for more than 80% of bariatric procedures.^17^ Sleeve gastrectomy is selectively performed for patients with body mass index (BMI; calculated as weight in kilograms divided by height in meters squared) of 60 or greater as part of a 2-stage duodenal switch procedure or when a gastric bypass is contraindicated for medical (eg, inflammatory bowel disease, need for certain medications) or surgical (eg, small bowel disease or adhesions) reasons.^18^

### Study Cohort

The exposed group consisted of all patients who underwent bariatric surgery from January 2010 to December 2016 in Ontario. The unexposed group was first created by excluding all individuals who were not eligible for surgery. Exclusions included non-Ontario residents and individuals with BMI of 35 or less (ie, class I obesity), age 70 years or older, history of cancer within 2 years, active substance abuse, who had accessed palliative care, were pregnant as of the index date, underwent previous solid organ transplantation (ie, liver, heart, lung), had active cardiac disease or major revascularization procedure within 6 months of index date, or had severe liver disease with ascites within 1 year of the index date. After excluding ineligible individuals, potential unexposed controls were selected from a linked family medicine database consisting of more than 500 000 patients and more than 400 physicians if they had at least 1 appointment at which eligibility for bariatric surgery could be established. Diabetes status for both groups was ascertained using a validated, administrative data case definition for diabetes.^19^ Multiple linked administrative databases were used to derive demographic, socioeconomic, and clinical variables for both groups (eAppendix 1 in the Supplement).

### Outcomes

The main study outcome was all-cause mortality. Secondary outcomes included cause-specific mortality, which was independently coded from death certificates by the Office of the Registrar General of Ontario. We classified the causes as cardiovascular, oncologic, other medical (ie, respiratory, gastrointestinal, infectious, and other), and external (ie, trauma and suicide). Cause of death data were available until December 31, 2017. Further secondary outcomes were several diabetes-relevant outcomes: a composite cardiovascular outcome (ie, cardiovascular mortality, nonfatal myocardial infarction, stroke, percutaneous coronary intervention, coronary artery bypass graft, transient ischemic stroke, deep vein thrombosis, or pulmonary embolism), a composite renal outcome (ie, new dialysis treatment or transplantation), and retinopathy.

### Matching Process

Matching was conducted using propensity score methods. Individuals were matched based on a propensity score created using all variables in eAppendix 2 in the Supplement.^20,21^ The lookback window for clinical confounders was 5 years previous to the index date, except for health care utilization, which was 1 year. To further ensure a sufficient match on important variables, matching criteria beyond the propensity score included hard-matching based on age (±3 years), sex, BMI (±3) at index date, and date of diabetes diagnosis (±12 months). The index date for the surgery group was the date of surgery. The index date for the control group was a date in the database at which they met criteria for bariatric surgery. Index dates for matched pairs were within 3 months of each other. Matching was completed in a greedy, nearest neighbor in a one-to-one fashion, sequentially without replacement.

### Statistical Analysis

Matching was assessed using standardized differences with an importance threshold of 0.10. Standardized differences were used owing to their insensitivity to sample size, and distributions of covariates were assessed using variance ratios.^20,21^ Unadjusted mortality rates were calculated for the entire follow-up for clinically relevant strata (age, sex, procedure type, BMI, duration of diabetes diagnosis) specified a priori. Unadjusted mortality outcomes were evaluated using χ^2^ statistic and 95% CIs. We modeled survival using Cox proportional hazard models. Follow-up time was from the index date until the end of follow-up or the primary outcome was reached. Adjusted analyses included all matching variables, the relevant clinical variables that were derived a priori (eTable 1 in the Supplement), and any unbalanced variables based on a standardized difference more than 0.10. For strata with fewer than 100 events, covariate adjustment was conducted using a propensity score and the treatment variable.^20^ Model assumptions were tested according to standard methods. *P* values were 2-tailed with statistical significance set at α = .05. Statistical analysis was performed using SAS statistical software version 9.4 (SAS Institute). Data were analyzed in 2020.

## Results

**Figure 1.**
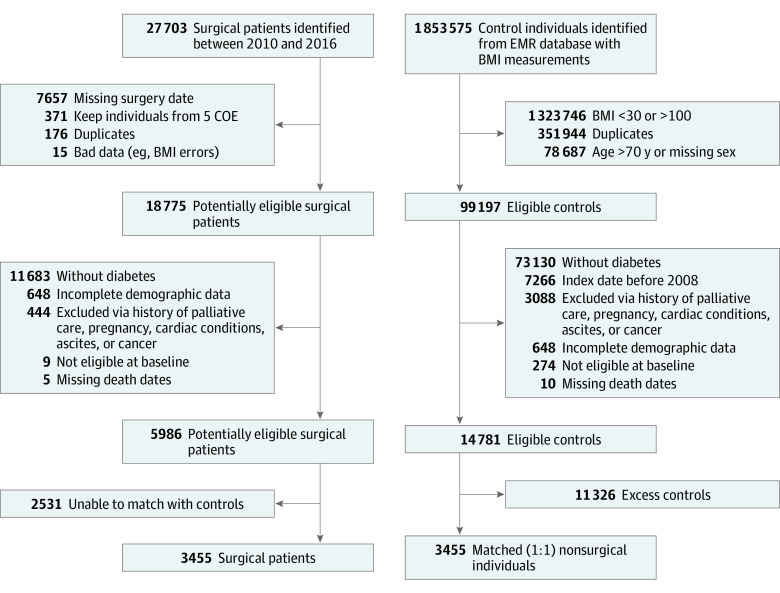
Flowchart of Cohort Creation and Identification of Eligible Patients for Inclusion BMI indicates body mass index (calculated as weight in kilograms divided by height in meters squared); COE, Centers of Excellence; and EMR, electronic medical record.

Figure 1 illustrates the participant recruitment flowchart. Table 1 shows the characteristics of study participants. In total, 3455 surgical patients were matched with 3455 controls. The mean (SD) age of the entire cohort was 52.04 (9.45) years, 4950 participants (71.6%) were women, and the mean (SD) BMI was 44.67 (7.9). Of 3455 patients who underwent bariatric surgery, 2994 (86.7%) underwent a gastric bypass. In terms of diabetes, 3054 participants (44.2%) were diagnosed within 5 years of the index date, and 712 participants (10.3%) had documented diabetes with microvascular or macrovascular complications. The control group was more likely to be from a rural area and to be active smokers, while the surgical group had a slightly higher prevalence of hypertension (Table 1). With respect to health care services utilization, the surgical group was more likely to have had at least 1 hospitalization, specialist visit, or colon cancer screening within 5 years. Participants in the control group had a higher rate of diabetic assessment and inpatient or hospital psychiatric assessment than the surgical group (Table 1).

**Table 1.** Baseline Characteristics of Study Participants

Characteristic	Individuals, No. (%)			Standardized difference^**a**^	Variance ratio
	Surgery (n = 3455)	Control (n = 3455)	Total (n = 6910)		
Age at baseline, mean (SD), y	51.66 (9.20)	52.41 (9.67)	52.04 (9.45)	0.08	0.91
BMI at baseline, mean (SD)	45.29 (7.55)	44.06 (8.25)	44.67 (7.94)	0.16	0.84
Sex					
Women	2475 (71.6)	2475 (71.6)	4950 (71.6)	0	1.00
Men	980 (28.4)	980 (28.4)	1960 (28.4)	0	1.00
Income quintile					
1	727 (21.0)	892 (25.8)	1619 (23.4)	0.11	1.15
2	735 (21.3)	787 (22.8)	1522 (22.0)	0.04	1.05
3	749 (21.7)	697 (20.2)	1446 (20.9)	0.04	0.95
4	689 (19.9)	612 (17.7)	1301 (18.8)	0.06	0.91
5	555 (16.1)	467 (13.5)	1022 (14.8)	0.07	0.87
Immigrant status	258 (7.5)	287 (8.3)	545 (7.9)	0.03	0.93
Rural status	596 (17.3)	699 (20.2)	1295 (18.7)	0.08	1.13
Smoking status	279 (8.1)	409 (11.8)	688 (10.0)	0.13	1.41
Procedure					
Gastric bypass	2994 (86.7)	NA	2994 (43.3)	3.6	0
Sleeve gastrectomy	461 (13.3)	NA	461 (6.7)	0.55	0
Diabetes history					
With microvascular or macrovascular complications	403 (11.7)	309 (8.9)	712 (10.3)	0.09	0.79
Duration, y					
≤5	1525 (44.1)	1529 (44.3)	3054 (44.2)	0	1.00
>5-10	939 (27.2)	947 (27.4)	1886 (27.3)	0.01	1.01
>10-15	576 (16.7)	572 (16.6)	1148 (16.6)	0	0.99
>15	415 (12.0)	407 (11.8)	822 (11.9)	0.01	0.98
Cardiovascular history					
Any cardiac disease	326 (9.4)	260 (7.5)	586 (8.5)	0.07	0.81
Heart failure	101 (2.9)	113 (3.3)	214 (3.1)	0.02	1.11
Stenting or CABG	213 (6.2)	152 (4.4)	365 (5.3)	0.08	0.73
Valve disease	12 (0.3)	7 (0.2)	19 (0.3)	0.03	0.58
MI	65 (1.9)	58 (1.7)	123 (1.8)	0.02	0.89
Atrial fibrillation	75 (2.2)	55 (1.6)	130 (1.9)	0.04	0.74
Other medical history					
Stroke	15 (0.4)	9 (0.3)	24 (0.3)	0.03	0.60
COPD	200 (5.8)	208 (6.0)	408 (5.9)	0.01	1.04
Hypertension	546 (15.8)	402 (11.6)	948 (13.7)	0.12	0.77
Sleep apnea	114 (3.3)	126 (3.6)	240 (3.5)	0.02	1.10
Renal disease	178 (5.2)	172 (5.0)	350 (5.1)	0.01	0.97
Dialysis	12 (0.3)	10 (0.3)	22 (0.3)	0.01	0.83
Liver disease	22 (0.6)	29 (0.8)	51 (0.7)	0.02	1.32
IBD	43 (1.2)	38 (1.1)	81 (1.2)	0.01	0.89
Previous malignant neoplasm^b^	47 (1.4)	44 (1.3)	91 (1.3)	0.01	0.94
Substance abuse	141 (4.1)	176 (5.1)	317 (4.6)	0.05	1.24
Alcohol	0	≤5 (0.1)	≤5 (<0.1)	0.02	1.12
Opioids	10 (0.3)	9 (0.3)	19 (0.3)	0.01	0.90
Cocaine	0	0	0	0	0
Eating disorder	≤5 (<0.1)	≤5 (<0.1)	8 (<0.1)	0.01	0
Mood disorder	53 (1.5)	66 (1.9)	119 (1.7)	0.03	1.24
Severe depression	≤5 (<0.1)	6 (0.2)	11 (0.2)	0.01	1.20
Schizophrenia	≤5 (<0.1)	25 (0.7)	27 (0.4)	0.11	12.42
Suicide or self-harm	≤5 (<0.1)	14 (0.4)	18 (0.3)	0.06	3.49
Medication	≤5 (<0.1)	12 (0.3)	16 (0.2)	0.05	2.99
Alcohol	≤5 (<0.1)	≤5 (<0.1)	≤5 (<0.1)	0	0
Chemical	0	≤5 (<0.1)	≤5 (<0.1)	0.03	0
Physical trauma	0	≤5 (<0.1)	≤5 (<0.1)	0.04	0
Health services utilization^c^					
Family physician visit	3365 (97.4)	3354 (97.1)	6719 (97.2)	0.02	1.12
Hospitalization	1687 (48.8)	1419 (41.1)	3106 (44.9)	0.16	0.97
ED visit	1691 (48.9)	1765 (51.1)	3456 (50.0)	0.04	1.00
Specialist visit	3455 (100.0)	3329 (96.4)	6784 (98.2)	0.28	0.97
Diabetic assessment	1517 (43.9)	1850 (53.5)	3367 (48.7)	0.19	1.01
Diabetic specialist visit	77 (2.2)	38 (1.1)	115 (1.7)	0.09	0.50
Cancer screening^b^					
Colon	1614 (46.7)	1227 (35.5)	2841 (41.1)	0.23	0.92
Cervical	1373 (39.7)	1383 (40.0)	2756 (39.9)	0.01	1.00
Breast	1280 (37.0)	1253 (36.3)	2533 (36.7)	0.02	0.99
Inpatient or hospital psychiatric assessment	≤5 (<0.1)	38 (1.1)	43 (0.6)	0.12	7.53
Form 1^d^	≤5 (<0.1)	27 (0.8)	28 (0.4)	0.12	26.80
Form 3^e^	0	≤5 (<0.1)	≤5 (<0.1)	0.05	0
Consultation for involuntary psychiatric treatment	0	≤5 (<0.1)	≤5 (<0.1)	0.02	0

Abbreviations: BMI, body mass index (calculated as weight in kilograms divided by height in meters squared); CABG, coronary artery bypass graft; COPD, chronic obstructive pulmonary disease; IBD, inflammatory bowel disease; MI, myocardial infarction; NA, not applicable.

^a^ Difference between sample means divided by pooled SD. Values greater than 0.1 are generally considered meaningful.

^b^ Within 5 years.

^c^ Within 1 year.

^d^ Application by Physician for Psychiatric Assessment. The Form 1 allows a physician to involuntarily hold patients in a psychiatric facility for up to 72 hours to undergo a psychiatric assessment.

^e^ Application by Physician for Psychiatric Assessment. The Form 3 allows a physician to involuntarily hold patients in a psychiatric facility for up to 2 weeks to undergo a psychiatric assessment.

**Figure 2.**
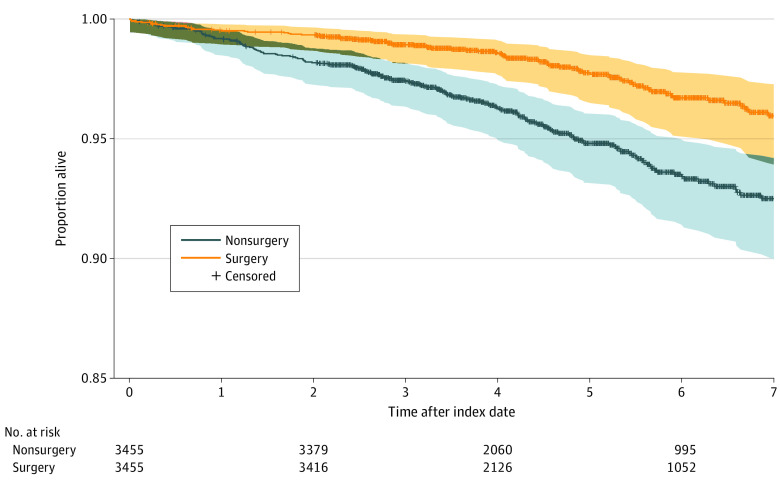
Kaplan-Meier Curves of All-Cause Mortality for Patients Who Underwent Bariatric Surgery and Matched Nonsurgical Controls

Table 2 illustrates the association between bariatric surgery and all-cause mortality overall and according to different patient characteristics (ie, sex, procedure type, diabetes duration, age, and BMI). Overall, there were 261 deaths (3.7%) during a median (interquartile range [IQR]) follow-up of 4.6 (3.22-6.35) years (Figure 2). There were 83 deaths (2.4%) in the surgery group and 178 deaths (5.2%) in the control group. The absolute risk reduction (ARR) for mortality associated with bariatric surgery was 2.7% (95% CI, 1.9-3.6%), and the adjusted hazard ratio (HR) for the surgery group was 0.53 (95% CI, 0.41-0.69). The absolute difference was larger in men (ARR, 3.7% [95% CI, 1.7%- to 5.7%]; HR, 0.56 [95% CI, 0.37-0.84]), although relative differences were similar for both sexes (women: ARR, 2.4 [95% CI, 1.4%-3.4%]; HR, 0.52 [95% CI, 0.37-0.73]). Patients who underwent gastric bypass had a 46% lower hazard of all-cause mortality compared with their matched counterparts (HR, 0.54 [95% CI, 0.40-0.71]). A similar association was observed in patients who received a sleeve gastrectomy. Overall, the observed associations of gastric bypass or sleeve gastrectomy with primary and secondary outcomes were similar.

**Table 2.** Association Between Bariatric Surgery and Mortality According to Sex, Procedure Type, Duration of Diabetes Diagnosis, Age, and BMI Among a Matched Cohort of Individuals With Type 2 Diabetes

**Characteristic**	Group	No.	Follow-up, median (IQR), y	Total deaths, No. (%)	ARR, % (95% CI)
Overall	Surgery	3455	4.67 (3.26 to 6.41)	83 (2.4)	2.7 (1.9 to 3.6)
	Control	3455	4.61 (3.17 to 6.30)	178 (5.2)	
Sex					
Men	Surgery	980	4.68 (3.24 to 6.37)	35 (3.5)	3.7 (1.7 to 5.7)
	Control	980	4.54 (3.24 to 6.21)	71 (7.2)	
Women	Surgery	2475	4.68 (3.27 to 6.44)	48 (1.9)	2.4 (1.4 to 3.4)
	Control	2475	4.63 (3.20 to 6.38)	107 (4.3)	
Procedure type					
Gastric bypass	Surgery	2994	4.78 (3.31 to 6.55)	70 (2.3)	2.9 (2.0 to 3.9)
	Control	2994	4.71 (3.20 to 6.48)	158 (5.3)	
Sleeve gastrectomy	Surgery	461	4.18 (3.08 to 5.57)	13 (2.8)	1.5 (−0.9 to 3.9)
	Control	461	4.10 (3.07 to 5.39)	20 (4.3)	
Duration of diabetes diagnosis, y					
≤5	Surgery	1525	4.68 (3.24 to 6.46)	23 (1.5)	2.2 (1.0 to 3.3)
	Control	1525	4.63 (3.17 to 6.46)	56 (3.7)	
>5-10	Surgery	939	4.77 (3.38 to 6.47)	20 (2.1)	3.1 (1.4 to 4.8)
	Control	939	4.73 (3.38 to 6.47)	49 (5.2)	
>10-15	Surgery	576	4.65 (3.22 to 6.30)	19 (3.3)	2.6 (0.2 to 5.0)
	Control	576	4.59 (3.10 to 6.05)	34 (5.9)	
>15	Surgery	415	4.56 (3.20 to 6.36)	21 (5.1)	4.3 (0.8 to 7.8)
	Control	415	4.41 (3.10 to 6.12)	39 (9.4)	
Age, y					
≤44	Surgery	749	4.70 (3.22 to 6.42)	8 (1.1)	0.5 (−0.6 to 1.7)
	Control	749	4.64 (3.18 to 6.35)	12 (1.6)	
45-54	Surgery	1247	4.66 (3.34 to 6.52)	23 (1.8)	1.8 (0.5 to 3.0)
	Control	1247	4.64 (3.25 to 6.46)	45 (3.6)	
≥55	Surgery	1459	4.70 (3.21 to 6.36)	52 (3.6)	4.7 (3.0 to 6.4)
	Control	1459	4.58 (3.10 to 6.24)	121 (8.3)	
BMI					
<40	Surgery	824	5.05 (3.44 to 6.75)	27 (3.3)	1.5 (−0.4 to 3.3)
	Control	824	4.89 (3.30 to 6.78)	39 (4.7)	
40-50	Surgery	1855	4.56 (3.18 to 6.20)	36 (1.9)	3.1 (1.9 to 4.3)
	Control	1855	4.43 (3.09 to 6.06)	94 (5.1)	
>50	Surgery	776	4.82 (3.33 to 6.65)	20 (2.6)	3.2 (1.2 to 5.2)
	Control	776	4.75 (3.33 to 6.48)	45 (5.8)	

Abbreviations: ARR, absolute risk reduction; BMI, body mass index (calculated as weight in kilograms divided by height in meters squared); IQR, interquartile range.

In terms of outcomes stratified by patient duration of diabetes, bariatric surgery was associated with reduced absolute mortality risk across all diabetic disease length cohorts and relative benefits for those with less than 15 years of diabetes. The association between surgery and survival was strongest in patients with disease duration of 5 years or less, with a 52% reduction in the hazard of mortality (HR, 0.48 [95% CI, 0.29-0.78]) and an ARR of 2.2% (95% CI, 1.0%-3.3%) at the end of follow-up. In patients with diabetes disease duration of more than 5 years to 10 years, bariatric surgery was associated with an ARR of 3.1% (95% CI, 1.4%-4.8%) and a decrease in hazard of all-cause mortality of 51% (HR, 0.49 [95% CI, 0.29-0.82]). Patients who had diabetes for longer than 15 years had an ARR of 4.3% (95% CI, 0.8%-7.8%), but the hazard of mortality was not statistically significantly reduced (HR, 0.66 [95% CI, 0.39-1.13]).

When stratified by different age groups, the benefit of bariatric surgery was strongest in patients aged 55 years or older, among whom there were 52 deaths (3.6%) in the surgical group compared with 121 deaths (8.3%) in matched controls. In this age group, hazard of all-cause mortality was reduced by 51%. Similarly, in individuals aged 45 to 54 years, surgery was associated with an ARR of 1.8% (95% CI, 0.5%-3.0%), and multivariate analysis revealed a 44% reduction in hazard of all-cause mortality (HR, 0.56 [95% CI, 0.34-0.93]). No mortality benefit of bariatric surgery was observed among those aged 44 years or younger at the end of follow-up, although the number of events in this group was low, with 20 deaths among 1498 individuals (1.3%). A sample size of approximately 8000 would be required to have adequate power to detect a mortality benefit in this age group. When stratified by BMI, patients with a BMI of 40 or greater experienced a mortality benefit with surgery. There were significant ARRs of 3.1% (95% CI, 1.9%-4.3%) in individuals with BMI 40 to 50 who underwent bariatric surgery compared with those who did not and 3.2% (95% CI, 1.2%-5.2%) in those with a BMI of greater than 50. Among individuals with BMI less than 40, there was no significant difference among those who underwent bariatric surgery and those who did not. Multivariable analysis demonstrated significant mortality benefit for patients with higher BMI, with a 52% reduction in all-cause mortality among those with a BMI of 40 to 50 (HR, 0.48 [95% CI, 0.32-0.70]) and a 56% in those with a BMI of greater than 50 (HR, 0.44 [95% CI, 0.26-0.74]) (eTable 2 in the Supplement). Patients with BMI less than 40 who underwent surgery had fewer deaths (27 deaths [3.3%] in the surgery group vs 39 deaths [4.7%] in the control group), but the difference was not significantly significant (HR, 0.79 [95% CI, 0.48-1.29]; *P* = .35).

Table 3 presents the associations of bariatric surgery with cause-specific mortality and composite cardiac, renal, and ophthalmic outcomes. Surgery was associated with reduced adjusted hazard of cardiac death (HR, 0.32 [95% CI, 0.15-0.66]) and cancer death (HR, 0.48 [95% CI, 0.26-0.91]) (Table 3; eFigure 1 in the Supplement). Furthermore, compared with the control group, the surgery group had a 34% reduction in hazard of the composite cardiovascular end point (HR, 0.68 [95% CI, 0.55-0.85]) and 42% reduction in hazard of the composite renal end point (HR, 0.58 [95% CI, 0.35-0.95]) (Table 3; eFigure 2 in the Supplement). There was no significant difference in nonfatal retinopathic outcomes. Overall, all curves met the proportional hazards assumption.

**Table 3.** Association Between Bariatric Surgery and Cause-Specific Mortality or Nonfatal Events in Patients With Diabetes

Event	No. of events^**a**^		Unadjusted HR		Adjusted HR^**b**^	
	Surgery (n = 3041)	Control (n = 3041)	HR (95% CI)	*P* value	HR (95% CI)	*P* value
Mortality						
Cardiac	9	38	0.23 (0.11-0.48)	<.001	0.32 (0.15-0.66)	.002
Cancer	14	33	0.42 (0.23-0.80)	.006	0.48 (0.26-0.91)	.02
Other medical	28	54	0.51 (0.32-0.80)	.003	0.60 (0.38-0.94)	.03
External (trauma or suicide)	6	13	0.45 (0.17-1.19)	.10	0.53 (0.20-1.41)	.21
Composite outcome						
Cardiovascular^c^	133	197	0.66 (0.53-0.82)	<.001	0.68 (0.55-0.85)	<.001
Renal^d^	24	43	0.55 (0.33-0.90)	.02	0.58 (0.35-0.95)	.03
Retinopathic outcome^e^	47	57	0.81 (0.55-1.19)	.28	0.81 (0.55-1.19)	.29

Abbreviation: HR, hazard ratio.

^a^ Includes individuals with cause of death data (available to December 2015).

^b^ Adjusted for age, body mass index, sex, immigrant status, income, rurality, diabetes status, overall cardiac history, stroke, chronic obstructive pulmonary disorder, hypertension, sleep apnea, renal disease, smoking status, previous malignant neoplasm, substance abuse, self-harm, mood disorder, cancer screening (colon, breast, cervical), and health care utilization in previous year (family physician, hospital inpatient, emergency department visit, specialist visit).

^c^ Cardiovascular composite included cardiac death, stroke, transient ischemic attack, myocardial infarction, percutaneous coronary intervention, coronary bypass grafting, new atrial fibrillation, pulmonary embolism, deep vein thrombosis; .

^d^ Renal composite outcomes included new dialysis or kidney transplant. Total sample size for renal events was 6038 individuals because we excluded individuals who previously experienced these events.

^e^ Total sample size for the retinopathic outcome was 5778 individuals because we excluded individuals who previously experienced retinopathy.

## Discussion

This cohort study presents one of the best-matched large cohorts of patients with diabetes examining the association of bariatric surgery with mortality in patients with diabetes, to our knowledge. Our study found that surgery was associated with an absolute reduction in mortality by 2.7% and a hazard reduction of all-cause mortality of 47%. In addition, surgery was associated with a 68% reduction in cardiac mortality and a 34% reduction in composite cardiac events. When stratified by age, BMI, sex, diabetes duration, and procedure type, surgery was associated with a pronounced absolute mortality benefit in men with diabetes and individuals aged 55 years or older. Importantly, surgery was associated with mortality benefits in most strata of patients with diabetes, and these results support current guidelines that bariatric surgery should be a first-line therapy for patients with diabetes and severe obesity.^22^ These findings suggest that among patients with diabetes, bariatric surgery was associated with observed benefits compared with a well-matched cohort. For younger patients and those with lower BMIs, longer studies may be required to examine associations with mortality.

The results of this study confirm the results of 2 cohort studies on bariatric surgery^23,24^ and has added to those studies by including important confounders, such as cancer screening. In a retrospective cohort study by Eliasson et al,^13^ 6132 patients with diabetes undergoing bariatric surgery were matched with controls. Eliasson et al^13^ reported a 58% reduction in mortality for patients with diabetes who underwent surgery compared with individuals who did not receive surgical treatment. Our study supports these results and strengthens the literature by adding a variety of additional confounders that help address bias, including utilization of health services, psychiatric history, and cancer screening. Our study also balances important confounders, such as heart failure, from that study. In another retrospective matched-cohort study by Aminian et al,^23^ a 41% reduction in mortality was reported among patients with diabetes who underwent bariatric surgery. Our study builds on the results by Aminian et al^23^ by including a larger cohort in a universal health care system. Our research group has previously investigated the association between bariatric surgery and all-cause mortality in patients with severe obesity using similar methods and a similar database.^23^ The previous study^23^ investigated all patients in Ontario, Canada, who received bariatric surgery (26.7% patients had diabetes), whereas this study specifically includes patients with type 2 diabetes only. Moreover, this study investigates nonfatal diabetes-related outcomes, such as cardiac, renal, and ophthalmic outcomes, which were not explored in the initial study. Our previous study^23^ did not show a significant mortality benefit associated with sleeve gastrectomy, but there was a significantly lower hazard of mortality noted in patients with diabetes. These findings underscore that patients with diabetes are an important subgroup in bariatric surgery with high potential benefits from surgery.

Reducing the risk of mortality is among the most important goals of diabetes treatment. However, studies have found that tight glycemic control from medical and lifestyle interventions offers no benefit toward all-cause mortality and cardiovascular mortality compared with standard glycemic control at 5 years of follow-up.^8,9^ Furthermore, drug trials for the treatment of diabetes report only modest improvements in the number of cardiovascular events and mortality, and none were geared solely toward patients who were obese.^24,25^ Therefore, while the medical management of diabetes can have modest benefits, the associations of many treatments with outcomes among patients with obesity is unknown. In contrast, the findings of this study suggest that bariatric surgery was associated with reduced all-cause mortality for patients with severe obesity and diabetes. Secondary to reducing mortality risk, preventing cardiovascular morbidity in patients is one of the major goals of diabetes management. Our findings demonstrate that bariatric surgery was associated with preventing cardiac events in patients with diabetes and obesity, likely through the synergistic interactions of weight loss, glycemic control, and other neurohumoral mechanisms for maintaining weight loss and glycemic control. Furthermore, patients in our study who underwent bariatric surgery had lower risk of nonfatal renal outcomes, which suggests an association of bariatric surgery with limiting noncardiac diabetic outcomes.

The most notable novel contribution of this study is the stratified analysis by patient characteristics, since it gives important insights into the mechanisms of the mortality benefit associated with bariatric surgery. The associations of surgery with outcomes were comparable in both sexes, patients aged 45 years and older, those with BMI of 40 or greater, and all diabetes duration. Specifically, in patients aged 55 years and older and men, bariatric surgery was associated with pronounced mortality benefit, thus suggesting that bariatric surgery should be aggressively recommended for patients within this population. This provides better understanding for obesity management in the primary care setting, since men are less likely to pursue surgery or adhere to bariatric surgery programs in a universal health care system.^26^ Moreover, although there is conflicting evidence^27,28,29^ surrounding older age as a risk factor for mortality in bariatric surgery, our study demonstrates that bariatric surgery was associated with substantial mortality benefit for older patients with diabetes over the medium-term (4.7 years). Thus, health care practitioners should continue to encourage surgery as an option to control diabetes and obesity for all men and older individuals. Although there was no statistically significant difference in risk of mortality in individuals with BMI less than 40 or aged 44 years or younger, this is likely from low event rates, and longer follow-up may be needed to study these cohorts. It could also be that mortality benefit associated with surgery is more modest, because these individuals are more likely to survive other comorbid events, such as myocardial infarctions. In addition, bariatric surgery in patients who had diabetes for more than 15 years was associated with mortality benefit, which supports the notion that the deleterious effects of diabetes may be substantially mitigated even when an individual has had the disease for an extended period of time.^30^ This study also underscores the need for equitable access to bariatric surgery. While access is available to all patients within the Ontario Bariatric Network, distance to centers may limit this, and previous studies, such as a 2016 study by Doumouras et al,^16^ have reported a significant regional variations in access. Accordingly, all regions should have access to this important treatment modality for patients with obesity and diabetes.

### Limitations

Our results should be interpreted in light of several limitations. There were differences in the baseline characteristics between the surgical and control groups. Although we adjusted for numerous known confounders, residual confounding by unmeasured factors is always possible in observational research. However, the amount of unmeasured bias would have to be substantial to mitigate the results. Second, while we strove to include all relevant clinical parameters in our model to account for all cofounding, there is a possibility of overfitting and collinearity within the model compared with the most statistically ideal model. Overall, we felt the inclusion of clinically important variables, some not seen in previous studies, outweighed the most ideal statistical model. Third, patients receiving bariatric surgery in Ontario undergo an extensive preoperative regimen of approximately 12 months.^26^ Therefore, patients who undergo surgery are likely to be more adherent to challenging postoperative lifestyle changes.^31^ Importantly, only 5% of patients eligible for surgery are referred for bariatric surgery,^26^ and this is mainly owing to it not being offered as opposed to patient choice,^32^ suggesting that there are sufficient healthy controls in the family medicine database. Furthermore, all patients had similar rates of family physician and specialist visits, and we matched on the propensity to seek preventive health care measures, such as cancer screening. This may mitigate some of the bias against patients who would not seek healthy behaviors.

## Conclusions

This cohort study found that among patients with diabetes and BMI of 35 or greater, bariatric surgery was associated with substantially lower risk of death across all important patient strata compared with nonsurgical management. Bariatric surgery also was associated with a lower risk of overall cardiac events and nonfatal renal outcomes. Overall, this study reinforces that the glycemic benefit of bariatric surgery found in randomized clinical trials likely translates to a mortality benefit over time, and it supports the use of surgery as a first-line treatment for individuals with obesity and diabetes.

## Notes

Supplement. **eAppendix 1.** Data Sources **eAppendix 2.** Potentially Confounding Variables Included in the Multivariate Regression Analysis **eTable 1.** Data Sources and Variable Definitions **eTable 2.** Adjusted Hazard Ratio of Mortality Stratified by Sex, Procedure Type, Duration of Diabetes Diagnosis, Age, and BMI **eFigure 1.** Cumulative Incidence Curves of Cause-Specific Mortality for Surgical Patients and Matched Nonsurgical Controls **eFigure 2.** Cumulative Incidence Curves of Adverse Outcomes for Surgical Patients and Matched Nonsurgical Controls
