# Error in Table 1

**DOI:** 10.1001/jamanetworkopen.2021.15446

**Published:** 2021-05-25

In the Original Investigation titled “Association Between Bariatric Surgery and Major Adverse Diabetes Outcomes in Patients With Diabetes and Obesity,”^1^ published April 26, 2021, some data in Table 1 were not masked in accordance with ICES policy. This article has been corrected.^1^

## References

[1] Doumouras AG, Lee Y, Paterson JM, . Association between bariatric surgery and major adverse diabetes outcomes in patients with diabetes and obesity. JAMA Netw Open. 2021;4(4):e216820. doi:10.1001/jamanetworkopen.2021.6820 33900401 PMC8076963

