# Supplementary material for: Association Between Bariatric Surgery and Major Adverse Diabetes Outcomes in Patients With Diabetes and Obesity
Source: JAMA Netw Open. 2021 Apr 26;4(4):e216820. doi: 10.1001/jamanetworkopen.2021.6820 (PMC8076963; doi:10.1001/jamanetworkopen.2021.6820)
Supplement: Supplement. — eAppendix 1. Data Sources eAppendix 2. Potentially Confounding Variables Included in the Multivariate Regression Analysis eTable 1. Data Sources and Variable Definitions eTable 2. Adjusted Hazard Ratio of Mortality Stratified by Sex, Procedure Type, Duration of Diabetes Diagnosis, Age, and BMI eFigure 1. Cumulative Incidence Curves of Cause-Specific Mortality for Surgical Patients and Matched Nonsurgical Controls eFigure 2. Cumulative Incidence Curves of Adverse Outcomes for Surgical Patients and Matched Nonsurgical Controls [file jamanetwopen-e216820-s001.pdf]

Supplemental Online Content

Doumouras AG, Lee Y, Paterson JM, et al. Association between bariatric surgery and major adverse diabetes outcomes in patients with diabetes and obesity. *JAMA Netw Open*. 2021;4(4):e216820. doi:10.1001/jamanetworkopen.2021.6820

eAppendix 1. Data Sources
eAppendix 2. Potentially Confounding Variables Included in the Multivariate Regression Analysis
eTable 1. Data Sources and Variable Definitions
eTable 2. Adjusted Hazard Ratio of Mortality Stratified by Sex, Procedure Type, Duration of Diabetes Diagnosis, Age, and BMI
eFigure 1. Cumulative Incidence Curves of Cause-Specific Mortality for Surgical Patients and Matched Nonsurgical Controls
eFigure 2. Cumulative Incidence Curves of Adverse Outcomes for Surgical Patients and Matched Nonsurgical Controls

This supplemental material has been provided by the authors to give readers additional information about their work.

© 2021 Doumouras AG et al. *JAMA Network Open.*

eAppendix 1. Data Sources

(1) Ontario Health Insurance Plan (OHIP) Registered Persons Database (RPDB), which captures information regarding all individuals covered by the publicly funded provincial health insurance plan, including date of birth, sex, place of residence, and date of death, if applicable.
(2) OHIP Claims History Database, which includes information pertaining to health services provided by Ontario physicians.
(3) CIHI DAD and CIHI National Ambulatory Care Reporting System (NACRS), which capture demographic, administrative, and clinical data regarding all acute hospital admissions and emergency department (ED) visits, respectively.
(4) Ontario Bariatric Registry (OBR), which houses all initial referral information for bariatric surgery patients in Ontario.
(5) Ontario Electronic Medical Record Administrative Linked Database (EMRALD), a database of linked primary care EMRs, which served as the data source for BMI for our matched comparison cohort.
(6) Immigration, Refugees and Citizenship Canada (IRCC) Permanent Resident Database, which provides information regarding the applicants and landing date of all immigrants who apply to land in Ontario.
(7) Office of the Registrar General of Ontario Death Register (ORGD), which tracks the date and cause of death for all Ontarians. These datasets were linked using unique coded identifiers and analyzed at ICES (www.ices.on.ca), an independent, non-profit research institute funded by an annual grant from the Ontario Ministry of Health and Long-Term Care (MOHLTC). As a prescribed entity under Ontario’s privacy legislation, ICES is authorized to collect and use health care data for the purposes of health system analysis, evaluation and decision support. Secure access to these data is governed by policies and procedures that are approved by the Information and Privacy Commissioner of Ontario.

© 2021 Doumouras AG et al. *JAMA Network Open.*

eAppendix 2. Potentially Confounding Variables Included in the Multivariate Regression Analysis

- Age
- Body Mass Index
- Sex
- Immigrant status
- Income quintile
- Rurality
- Diabetes status
- Overall cardiac history
    - Cardiac disease
    - Heart failure
    - Stenting/coronary artery bypass graft
    - Valve disease
    - Myocardial infarction
    - Atrial fibrillation
- Stroke
- Chronic obstructive pulmonary disease
- Hypertension
- Sleep apnea
- Renal disease
- Liver disease
- Inflammatory bowel disease
- Smoking status
- Previous malignancy
- Substance abuse
    - Alcohol
    - Opioids
    - Cocaine
- Self-harm or suicide
    - Medication
    - Alcohol
    - Chemical
    - Physical trauma
- Mood disorder
    - Severe depression
    - Schizophrenia
- Healthcare utilization in previous 1 year
    - Family physician visit
    - Hospitalization (inpatient)
    - Emergency department visit
    - Specialist visit
    - Diabetic assessment
    - Diabetic specialist visit
- Inpatient or hospital psychiatric assessment
    - Form 1 assessment
    - Form 3 assessment
    - Consultation for involuntary psychiatric treatment
- Cancer screening in previous 5 years
    - Colon cancer
    - Breast cancer
    - Cervical cancer

© 2021 Doumouras AG et al. *JAMA Network Open.*

eTable 1. Data Sources and Variable Definitions

Variable Name	Source	Type	Description
Age	RPDB	Registry	At index date
Sex	RPDB	Registry	N/A
Diabetic Status	ODD	Registry	2002-2017
Cancer Status	OCR	Registry	
Income Quintile	RPDB	Registry	N/A
Rurality	RPDB	Registry	N/A
Type of Bariatric procedure	OBR	Registry	2002-2017
Immigrant indicator	IRCC	Registry	2002-2017
LHIN	RPDB	Registry	2002-2017
Centre for Excellence	RPDB	Registry	2002-2017
Diabetes Duration	ODD	Registry	N/A
Heart Failure	DAD	Administrative	Dx10: I50
Stenting/CABG	DAD, OHIP	Administrative, Physician Service Claims	Incode: 1IJ50 1IJ76 Dx10: I20 I25<br>Feecode: Z434 R742 R743
Valve Disease	DAD, OHIP	Administrative, Physician Service Claims	Incode: 1HS80 1HS90 1HT80 1HT90 1HU80 1HU90 1HV80 1HV90 Dx10: I34 I35 I36 I37<br>Feecode: R930 R738 R863 R876 R729 R730 R734 R735 R733 R773 R774 R724 R725 R772 Z448
MI	DAD		Dx10: I22 I23 Incode: 1IL35
AFiB	DAD, OHIP	Administrative, Physician Service Claims	Dx10: I48<br>R753 R761 G317 G303 Z433 Z444 Z445 Z435 Z436
Stroke	DAD	Administrative	Dx10: I60 I61 I62 I63 I64 I65 I66
COPD	DAD	Administrative	Dx10: J43 J44
Hypertension	HYPER	Validated Administrative Cohort	2002-2017
Sleep Apnea	DAD	Administrative	Dx10: G473 G4738
Renal	DAD OHIP	Administrative, Physician Service Claims	Dx10: N18 N19 N052 N054 N055 N056 N057 N250 I120 I131 N032 N033 N034 N035 N036 N037 Z490 N8031 N8032 N8033 N8034 N8035 N8038 N8039 Z490 Z491 Z492 Z940 N1023 N1123 N1323 N1423<br><br>Dx: 304 585
Renal dialysis	DAD, OHIP	Administrative, Physician Service Claims	Incode: 1PZ21HQBR 1PZ21HPD4<br>Feecode: R849 R850 G323 G325 G326 G330 G331 G860 G333 G083 G091 G085 G295 G082 G090 G092 G093 G094 G861 G862 G863 G864 G865 G866 G294 G095 G096
IBD	DAD, OCCC	Administrative, Registry	R50 R51
COPD	COPD	Registry	2002-2017
Smoking status	DAD, OHIP	Administrative, Physician Service Claims	Dx10: F17 Z720 Z716 Z8643<br>Dx: 305 E079 K039 Q042 Q41 Q622
Previous Malignancy	OCR	Registry	Any previous malignancy within the Ontario Cancer Registry
Alcohol	DAD, OHIP	Administrative, Physician Service Claims	Dx10: F10 Z721 Z714 Z8640<br>Dx 291 303 571
Drugs	DAD, OHIP	Administrative, Physician Service Claims	Dx10: Z722 Z8641 Z715 F12 F13 F15 F16 F18 F19<br>Dx: 292 304
Opioids	DAD, OHIP	Administrative, Physician Service Claims	Dx10: F11<br>Dx: K682 K683 K684
Cocaine	DAD	Administrative	Dx10: F14
Eating disorder	DAD	Administrative	Dx10: F50

© 2021 Doumouras AG et al. *JAMA Network Open.*

Mood disorder	DAD	Administrative	Dx10: F30 F31 F32 F33 F34 F38 F39 F530
Severe depression	DAD	Administrative	Dx10: F322 F323 F3332 F333
Schizophrenia	DAD	Administrative	Dx10: F20 F21 F22 F23 F24 F25 F28 F29 F531
Medications	DAD	Administrative	Dx10: X61 X62 X63 X64
Alcohol	DAD	Administrative	Dx10: X65
Chemicals	DAD	Administrative	Dx10: X66 X67 X68 X69
Physical trauma	DAD	Administrative	Dx10: X70 X71 X72 X73 X74 X75 X76 X77 X78 X79 X80 X81 X82 X83 X84
Family doctor	OHIP	Physician Service Claims	Spec: 00
Hospitlization	DAD	Administrative	N/A
Emergency Room Visit	NACRS	Administrative	2006-2017
Specialist visit	OHIP	Physician Service Claims	Spec: 01-99
Diabetic assessment	OHIP	Physician Service Claims	Feecode: K030 Q040
Diabetic specialist	OHIP	Physician Service Claims	Feecode: K045 K046
Colon cancer	DAD, OHIP	Administrative, Physician Service Claims	Incode: 2NK70 2NK71 2NM70 2NM71 Feecode: G004 Z555 Z498 Z499 Z492 Z493 Z494 Z496 Z498 Z495 Z491
Cervical cancer	OHIP	Physician Service Claims	Feecode: G365 E430 G394 E431 Z731 Z787 Z730
Breast cancer	OHIP	Physician Service Claims	Feecode: X184 X185 X172 X178 J863
Form 1	OHIP	Physician Service Claims	Feecode: K623
Form 3	OHIP	Physician Service Claims	Feecode: K624
Consulation for involuntary psychiatric treatment	OHIP	Physician Service Claims	Feecode: K620

(1) Ontario Health Insurance Plan (OHIP) Claims History Database, which includes information pertaining to health services provided by Ontario physicians;
(2) the OHIP Registered Persons Database (RPDB), which provides information about all individuals registered for OHIP, such as their health insurance status date of birth, sex, location of residence and vital status;
(3) the CIHI-Discharge Abstract Database and CIHI National Ambulatory Care Reporting System (NACRS), national databases comprised of demographic, administrative, and clinical data for all hospital discharges and emergency department (ED) visits;
(4) the Ontario Bariatric Registry (OBR), which houses all initial referral information for bariatric surgery in Ontario;
(5) the Electronic Medical Record Administrative Linked Database (EMRALD), a database of family physician records throughout the province21,22;
(6) the Immigration, Refugees and Citizenship Canada (IRCC) Permanent Resident Database, which provides information regarding the applicants and landing date of all immigrants who apply to land in Ontario;
(7) the Ontario Registrar General Death Register (ORGD), which tracks all deaths and cause of death within the province;
(8) and the Ontario Diabetes Database (ODD), a population-based disease registry constructed using a validated algorithm based on hospitalizations and physician visits to identify individuals with physician-diagnosed diabetes mellitus in Ontario
(9) OCCC, HYPER, COPD, population-based disease registries constructed using a validated algorithm based on hospitalizations and physician visits to identify individuals with physician-diagnosed IBD, hypertension and COPD in Ontario

© 2021 Doumouras AG et al. *JAMA Network Open.*

eTable 2. Adjusted Hazard Ratio of Mortality Stratified by Sex, Procedure Type, Duration of Diabetes Diagnosis, Age, and BMI

	Arm	N	Person years followed	Mortality/1000 person years	IRR (95%CI)	Adjusted HR* (95%CI)	*P*
Overall	Surgery	3455	16,984.03	4.88	5.77 (3.88 – 7.65)	0.53 (0.41 – 0.69)	<0.001
	Control	3455	16,708.78	10.7			
Sex							
Males	Surgery	980	4,813.34	7.27	7.95 (3.67 – 12.23)	0.56 (0.37-0.84)	0.005
	Control	980	4,664.87	15.3			
Females	Surgery	2475	12,170.69	3.94	4.94 (2.92 – 6.96)	0.52 (0.37 – 0.73)	<0.001
	Control	2475	12,043.91	8.89			
Procedure Type							
Gastric Bypass	Surgery	2994	14,936.89	4.69	6.07 (4.06 – 8.07)	0.54 (0.40 – 0.71)	<0.001
	Control	2994	14,692.44	10.8			
Sleeve Gastrectomy	Surgery	461	2,047.14	6.35	3.57 (-1.98 – 9.12)	0.58 (0.29 – 1.17)	0.13
	Control	461	2,016.34	9.92			
Duration of diabetes diagnosis							
<5-year diabetics	Surgery	1525	7508.07	3.06	4.46 (2.13 – 6.80)	0.48 (0.29 – 0.78)	0.003
	Control	1525	7438.72	7.53			
5-10-year diabetics	Surgery	939	4693.63	4.26	6.36 (2.85 – 9.87)	0.49 (0.29-0.82)	0.007
	Control	939	4613.95	10.6			
10-15-year diabetics	Surgery	576	2802.40	6.78	5.61 (0.45 – 10.77)	0.54 (0.31 – 0.95)	0.03
	Control	576	2743.70	12.4			
>15-year diabetics	Surgery	415	1979.94	10.6	9.79 (1.94 – 17.63)	0.66 (0.39 – 1.13)	0.13
	Control	415	1912.40	20.4			
Age							
≤44 years	Surgery	749	3,697.05	2.16	1.11 (-1.28 – 3.49)	0.76 (0.39 – 1.85)	0.54
	Control	749	3,669.71	3.27			
45-54 years	Surgery	1,247	6,167.74	3.73	3.65 (1.01 – 6.29)	0.56 (0.34 – 0.93)	0.02
	Control	1,247	6,099.80	7.38			
≥55 years	Surgery	1,459	7,119.24	7.30	10.13 (6.45 – 13.8)	0.49 (0.35 – 0.68)	<0.001
	Control	1,459	6,939.27	17.4			
Body Mass Index (BMI)							
BMI ≤40 kg/m^2	Surgery	824	4,203.88	6.42	2.94 (-0.87 – 6.75)	0.79 (0.48 – 1.29)	0.35
	Control	824	4,165.31	9.36			
BMI 40-50 kg/m^2	Surgery	1855	8,879.61	4.05	6.76 (4.2 – 9.32)	0.48 (0.32 – 0.70)	<0.001
	Control	1855	8,691.70	10.8			
BMI ≥50 kg/m^2	Surgery	776	3,900.54	5.13	6.56 (2.47 – 10.64)	0.44 (0.26 – 0.74)	0.002
	Control	776	3,851.77	11.68			

CI, confidence interval; ARR; absolute risk reduction; IRR, incidence rate ratio; HR, hazard ratio

*Adjusted for age, sex, BMI, immigrant status, income quintile, rurality, history cardiac disease, stroke, COPD, hypertension, sleep apnea, renal disease, smoking, previous malignancy, substance abuse, self-harm, mood disorder, cancer screening (colon, breast, cervical) and healthcare utilization (encounters with family physicians, specialists, emergency departments, and hospitals) in the previous year

© 2021 Doumouras AG et al. *JAMA Network Open.*

eFigure 1. Cumulative Incidence Curves of Cause-Specific Mortality for Surgical Patients and Matched Nonsurgical Controls

(A) Cumulative incidence curves of cardiovascular mortality for surgical patients and matched nonsurgical patients

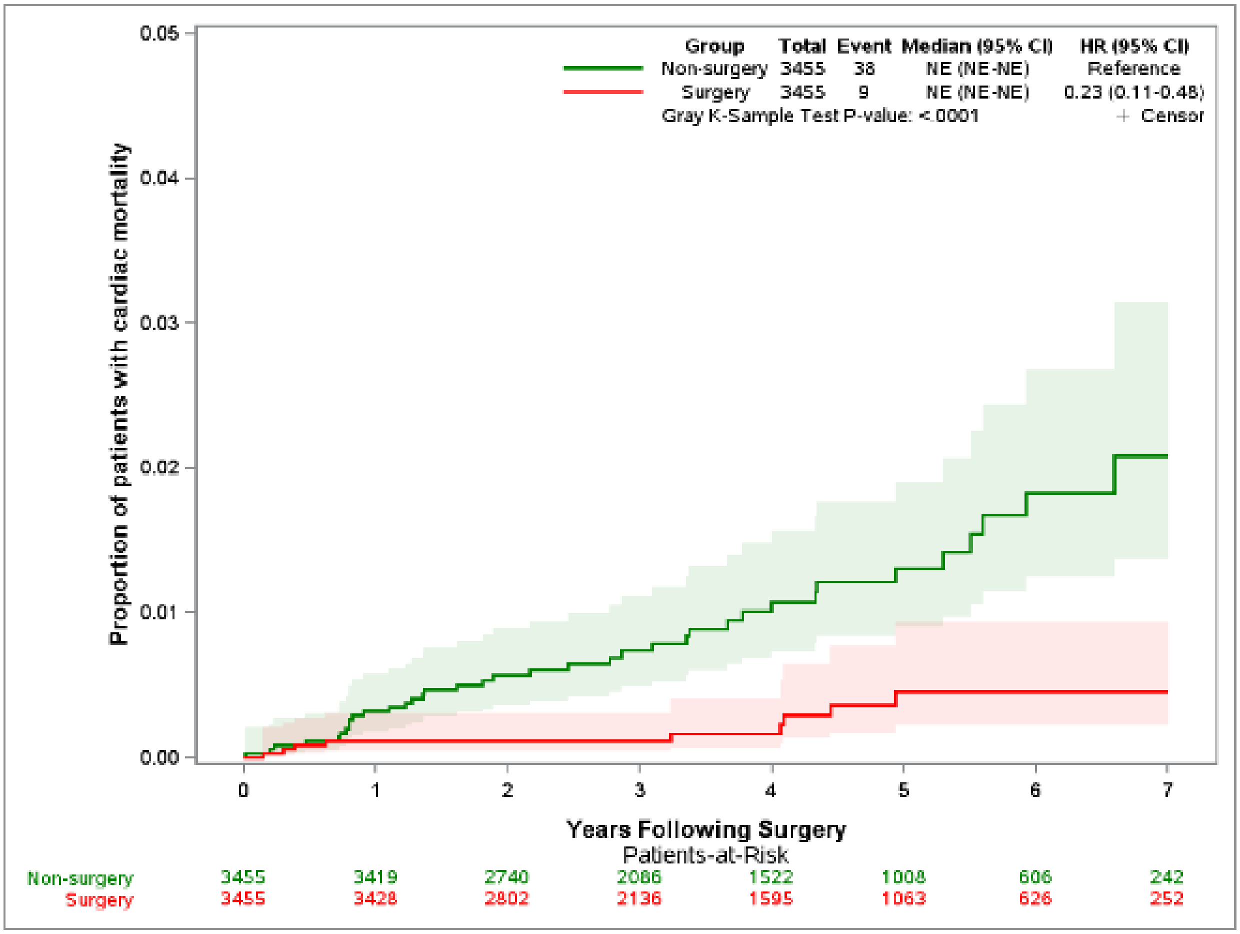

© 2021 Doumouras AG et al. *JAMA Network Open.*

eFigure 1. (B) Cumulative incidence curves of cancer mortality for surgical patients and matched nonsurgical patients

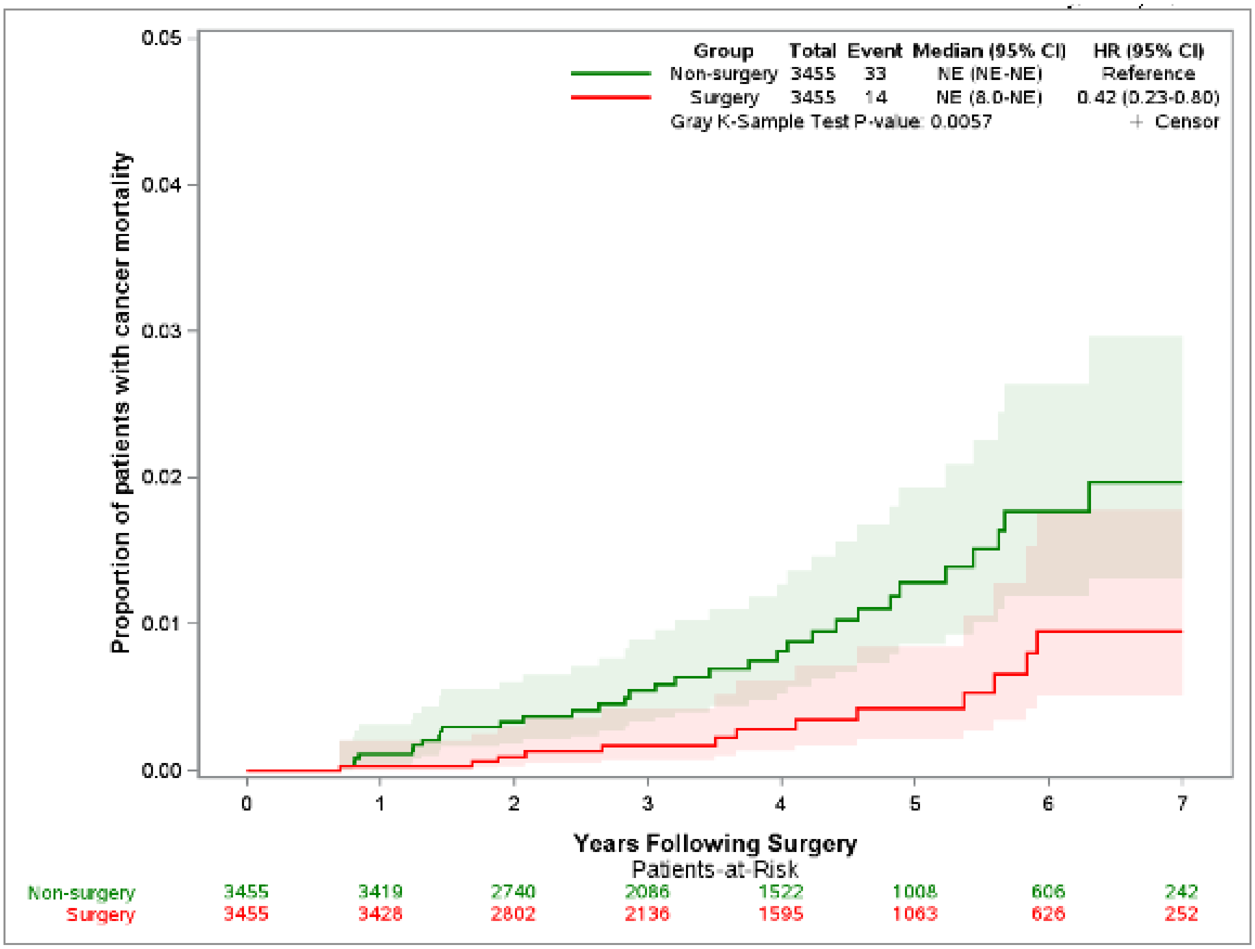

© 2021 Doumouras AG et al. *JAMA Network Open.*

eFigure 1. (C) Cumulative incidence curves of other mortality for surgical patients and matched nonsurgical patients

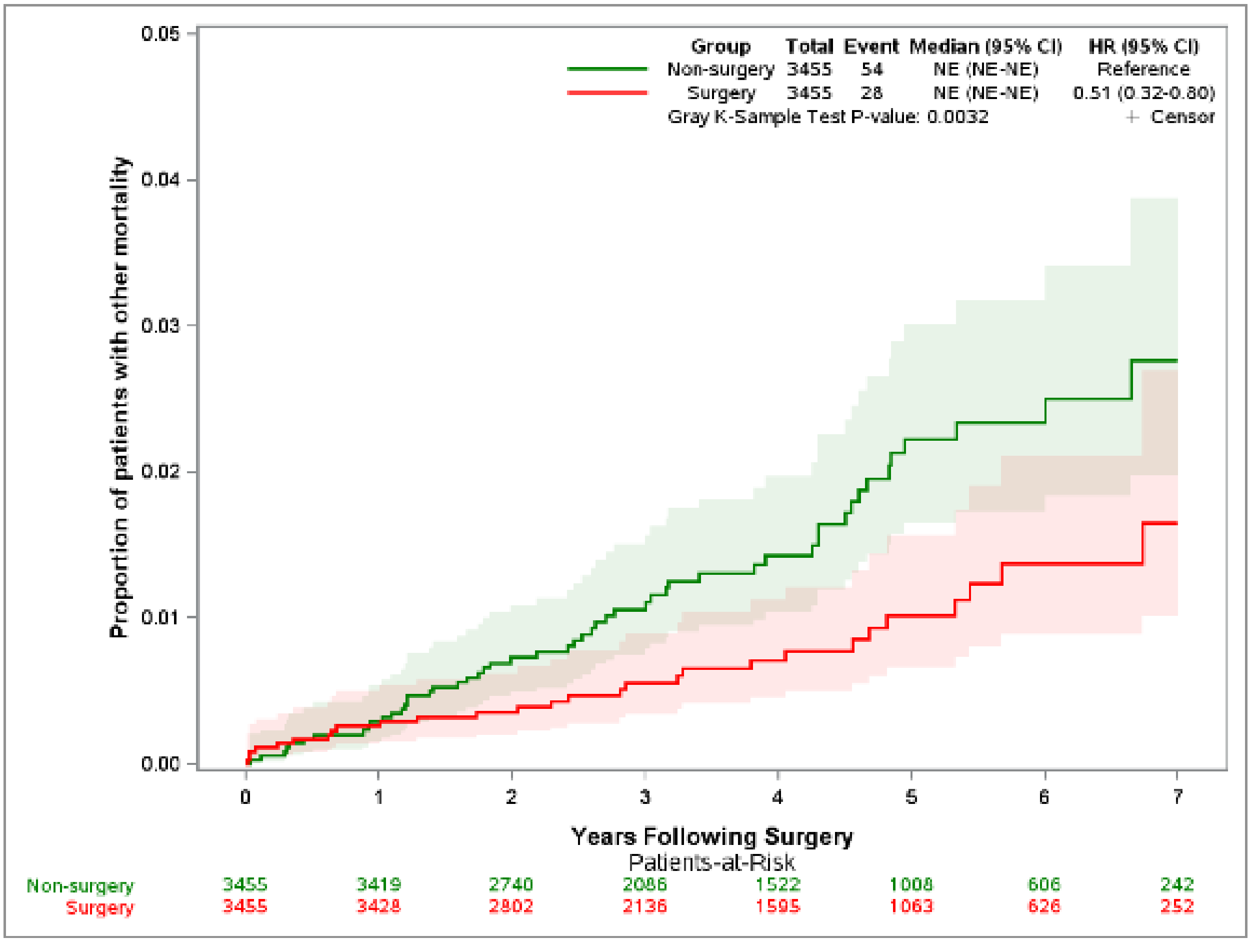

© 2021 Doumouras AG et al. *JAMA Network Open.*

eFigure 1. (D) Cumulative incidence curves of external mortality for surgical patients and matched nonsurgical patients.

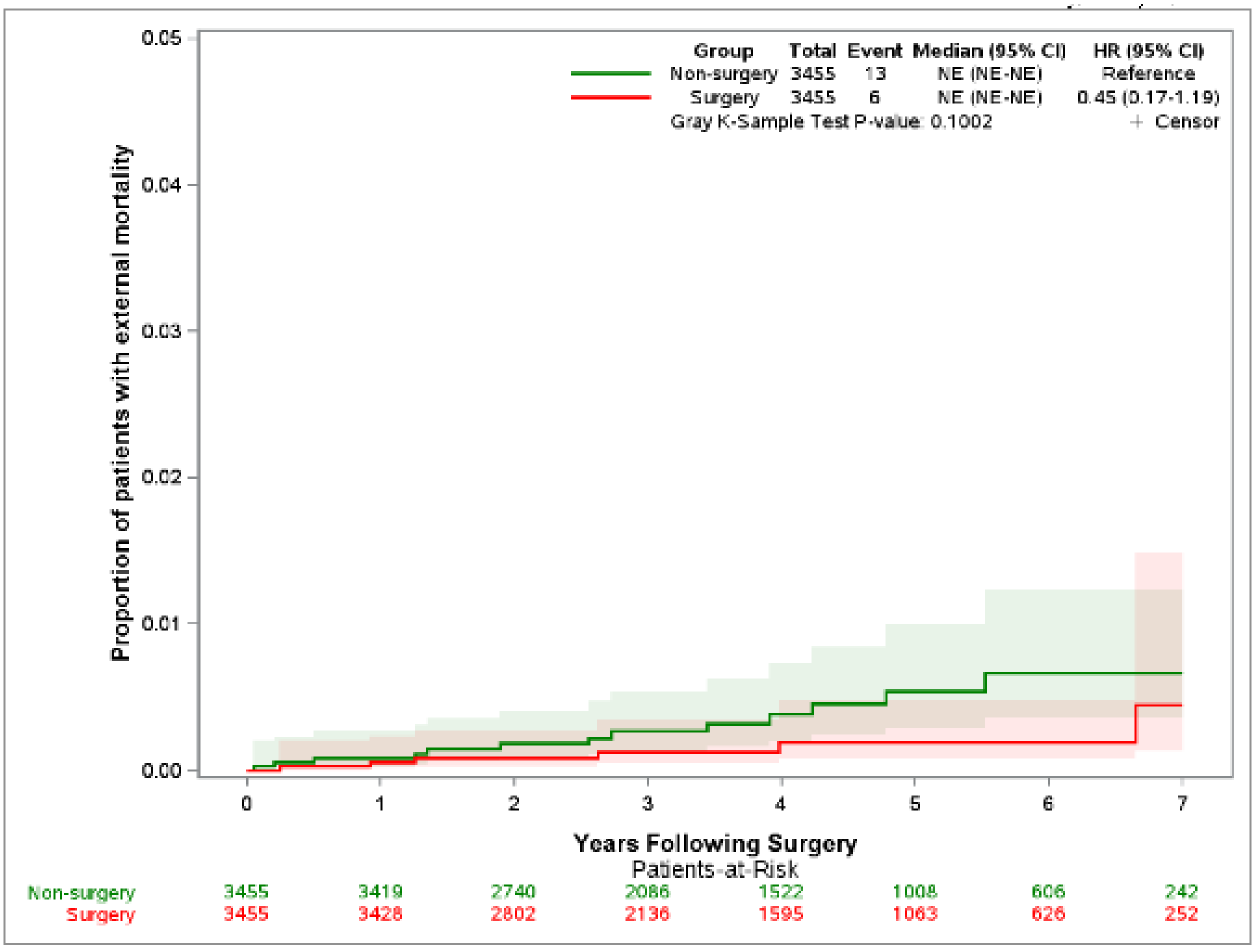

© 2021 Doumouras AG et al. *JAMA Network Open.*

eFigure 2. Cumulative Incidence Curves of Adverse Outcomes for Surgical Patients and Matched Nonsurgical Controls

(A) Cumulative incidence curves of overall cardiovascular outcomes for surgical patients and matched nonsurgical patients

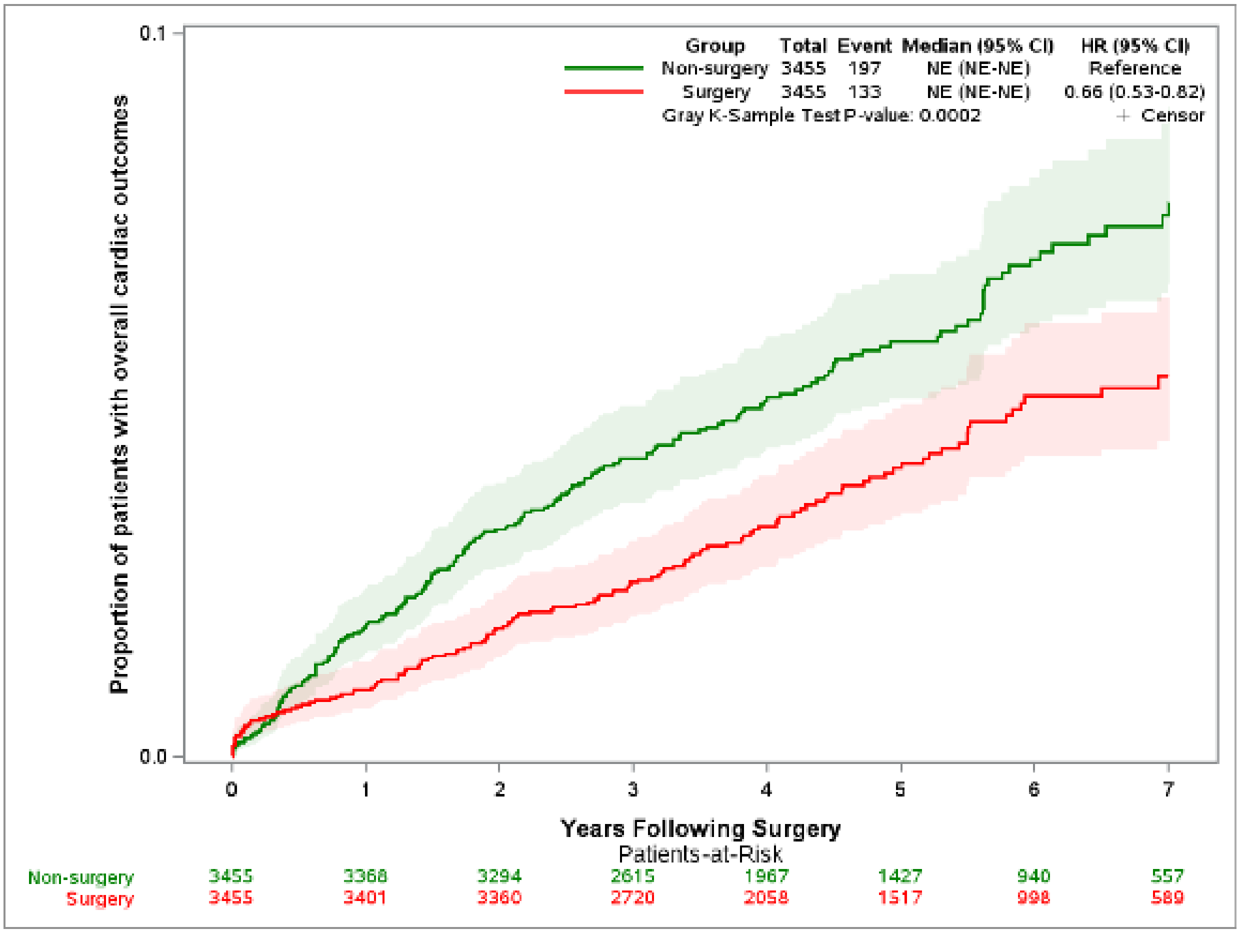

© 2021 Doumouras AG et al. *JAMA Network Open.*

eFigure 2. (B) Cumulative incidence curves of renal for surgical patients and matched nonsurgical patients

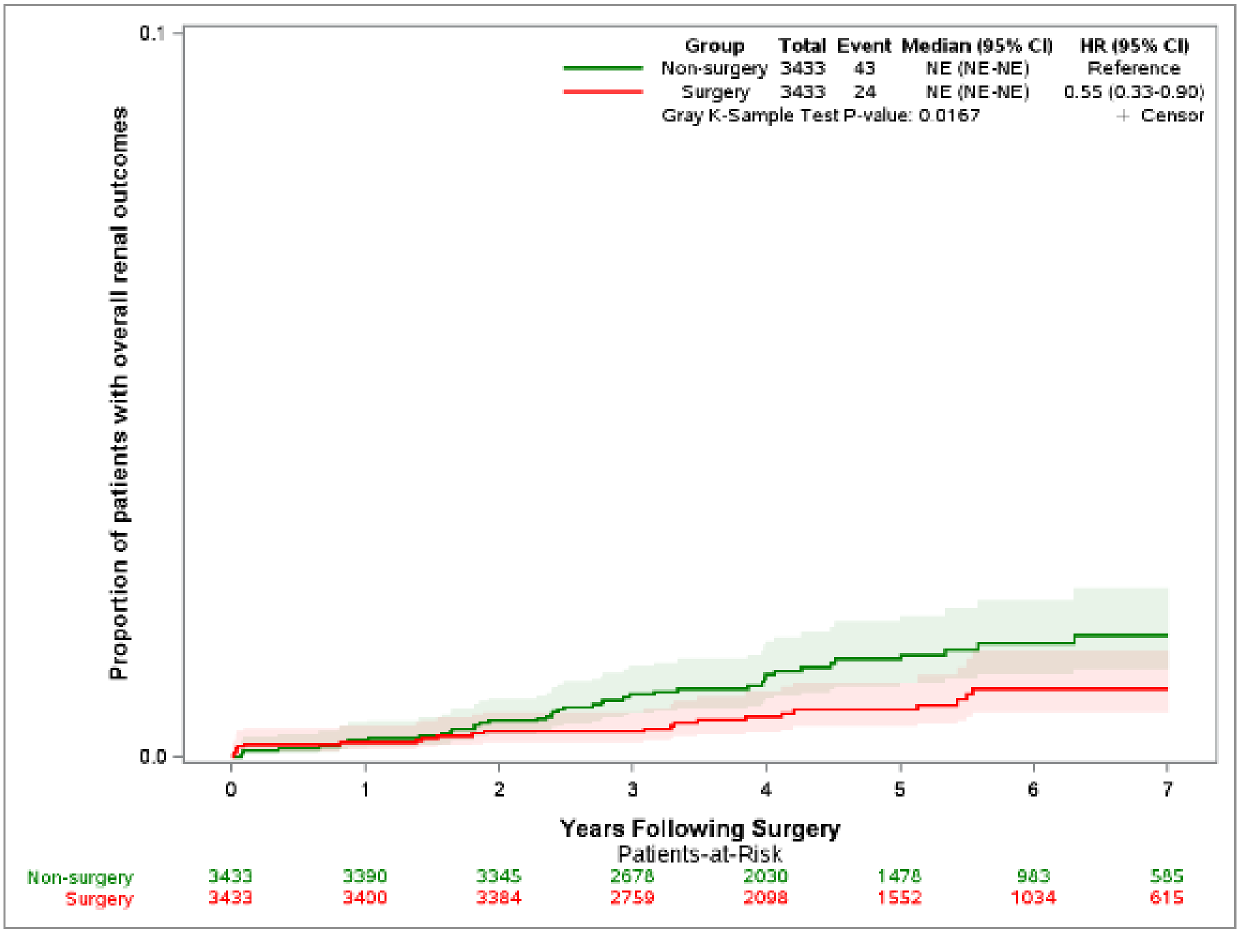

© 2021 Doumouras AG et al. *JAMA Network Open.*

eFigure 2. (C) Cumulative incidence curves of retinopathy for surgical patients and matched nonsurgical patients

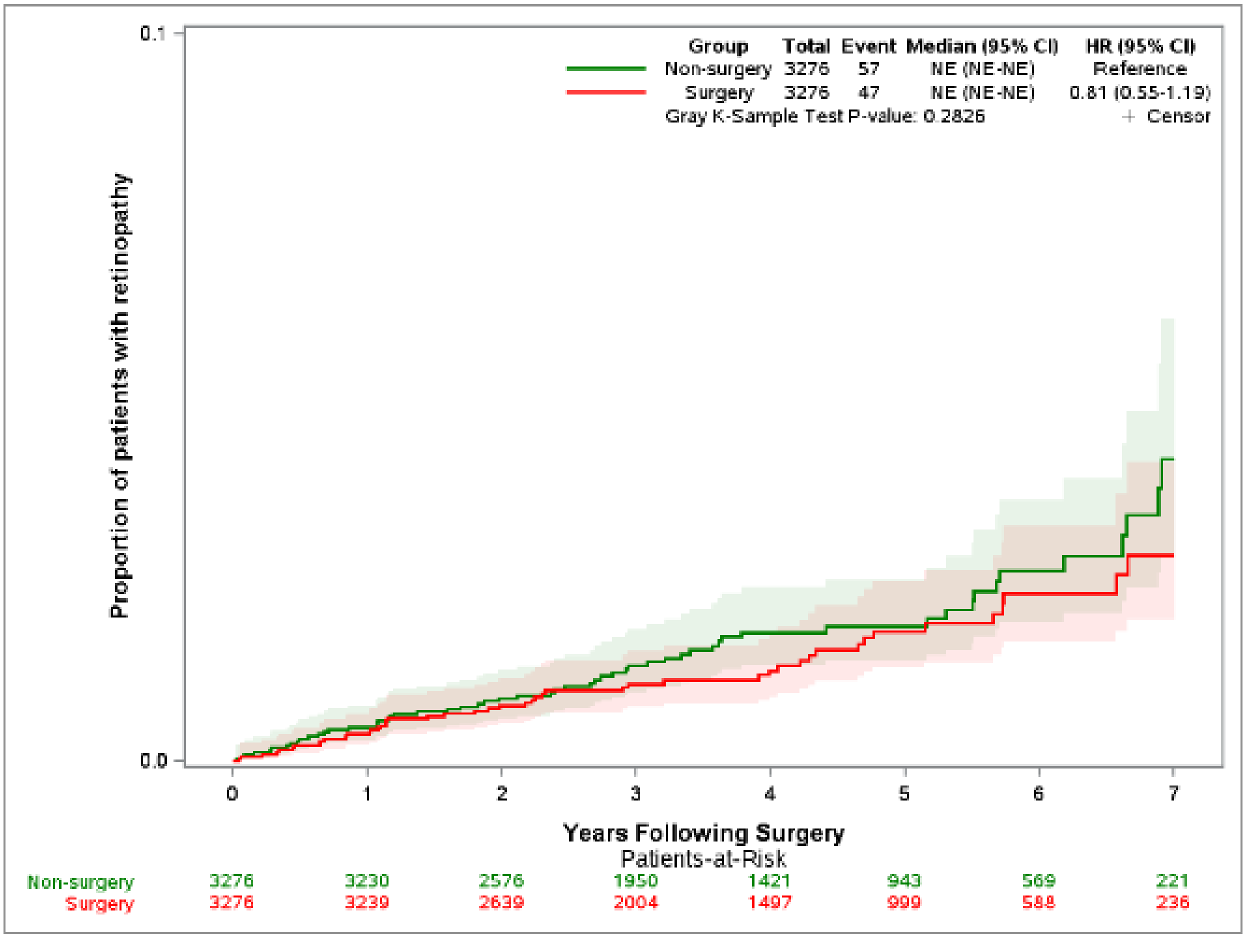

© 2021 Doumouras AG et al. *JAMA Network Open.*
